# *N*,*N*′-Bis(phenyl­sulfon­yl)maleamide

**DOI:** 10.1107/S1600536809053811

**Published:** 2009-12-24

**Authors:** B. Thimme Gowda, Sabine Foro, P. A. Suchetan, Hartmut Fuess

**Affiliations:** a Department of Chemistry, Mangalore University, Mangalagangotri 574 199, Mangalore, India; b Institute of Materials Science, Darmstadt University of Technology, Petersenstrasse 23, D-64287 Darmstadt, Germany

## Abstract

Mol­ecules of the title compound, C_16_H_14_N_2_O_6_S_2_, show crystallographic inversion symmetry: there is one half-mol­ecule in the asymmetric unit. The structure exhibits both intramolecular and inter­molecular N—H⋯O hydrogen bonds.

## Related literature

For our studies of the effect of ring and the side-chain substituents on the solid-state structures of *N*-aromatic sulfonamides, see: Gowda *et al.* (2009 ▶, 2010 ▶), Suchetan *et al.* (2009 ▶). 
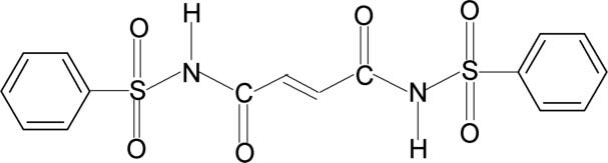


## Experimental

### 

#### Crystal data

C_16_H_14_N_2_O_6_S_2_
*M* *_r_* = 394.41
Monoclinic, 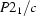

*a* = 8.582 (1) Å
*b* = 5.1464 (6) Å
*c* = 19.691 (4) Å
β = 101.17 (2)°
*V* = 853.2 (2) Å^3^
*Z* = 2
Mo *K*α radiation
μ = 0.35 mm^−1^
*T* = 299 K
0.48 × 0.28 × 0.22 mm

#### Data collection

Oxford Diffraction Xcalibur diffractometer with a Sapphire CCD detector
Absorption correction: multi-scan (*CrysAlis RED*; Oxford Diffraction, 2009 ▶) *T*_min_ = 0.850, *T*_max_ = 0.927
3236 measured reflections
1720 independent reflections
1500 reflections with *I* > 2σ(*I*)
*R*_int_ = 0.009

#### Refinement

*R*[*F* ^2^ > 2σ(*F* ^2^)] = 0.030
*wR*(*F* ^2^) = 0.083
*S* = 1.07
1720 reflections
121 parameters
1 restraint
H atoms treated by a mixture of independent and constrained refinement
Δρ_max_ = 0.25 e Å^−3^
Δρ_min_ = −0.32 e Å^−3^

### 

Data collection: *CrysAlis CCD* (Oxford Diffraction, 2009 ▶); cell refinement: *CrysAlis RED* (Oxford Diffraction, 2009 ▶); data reduction: *CrysAlis RED*; program(s) used to solve structure: *SHELXS97* (Sheldrick, 2008 ▶); program(s) used to refine structure: *SHELXL97* (Sheldrick, 2008 ▶); molecular graphics: *PLATON* (Spek, 2009 ▶); software used to prepare material for publication: *SHELXL97*.

## Supplementary Material

Crystal structure: contains datablocks I, global. DOI: 10.1107/S1600536809053811/bt5141sup1.cif

Structure factors: contains datablocks I. DOI: 10.1107/S1600536809053811/bt5141Isup2.hkl

Additional supplementary materials:  crystallographic information; 3D view; checkCIF report

## Figures and Tables

**Table 1:** Hydrogen-bond geometry (Å, °)

*D*—H⋯*A*	*D*—H	H⋯*A*	*D*⋯*A*	*D*—H⋯*A*
N1—H1*N*⋯O2^i^	0.84 (1)	2.35 (2)	3.0254 (19)	138 (2)
N1—H1*N*⋯O1^ii^	0.84 (1)	2.45 (2)	3.1335 (19)	139 (2)

Symmetry codes: (i) 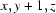; (ii) 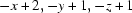.

**Fig. 1.**
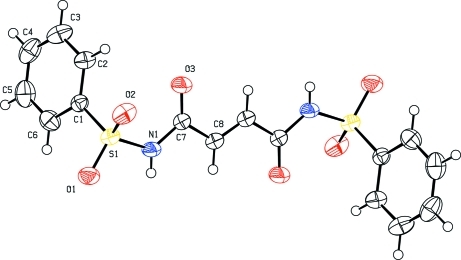
Molecular structure of (I), showing the atom labelling scheme and displacement ellipsoids are drawn at the 50% probability level.

**Fig. 2.**
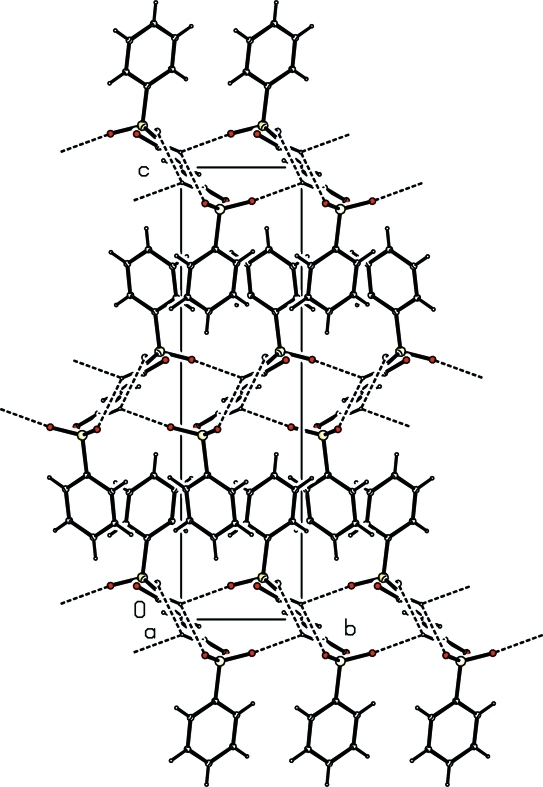
Molecular packing of (I) with hydrogen bonding shown as dashed lines.

## supplementary crystallographic information

### Comment

Diaryl acylsulfonamides are known as potent antitumor agents against a broad spectrum of human tumor xenografts in nude mice. As part of a study of the effect of ring and the side chain substituents on the solid state structures of *N*-aromatic sulfonamides (Gowda *et al.*, 2009, 2010; Suchetan *et al.*, 2009), in the present work, the structure of *N*,*N*-(Diphenylsulfonyl)maleamide (I) has been determined (Fig. 1).

The conformations of N—H and C=O bonds in the amide fragments are *trans* to each other and the amide O atoms are *anti* to the H atoms attached to the adjacent C atoms, similar to that observed in *N*,*N*-(diphenylsulfonyl)succinamide (II)(Gowda *et al.*, 2010). The molecule is bent at the S atoms with the C—SO_2_—NH—C(O) torsion angle of 66.1 (2)° in (II), compared to the value of 65.2 (2)°. The dihedral angle between the benzene ring and the SO_2_—NH—C(O)—C segment in the two halves of the molecule is 76.4 (1)°, compared to the corresponding angle of 77.4 (1)° in (II). The structure exhibits both the intramolecular and intermolecular hydrogen bonds. The series of N—H···O(S) hydrogen bonds (Table 1) link the molecules into column like infinite chains parallel to *a*-axis (Fig. 2).

### Experimental

*N*,*N*-(Diphenylsulfonyl)maleamide was prepared by refluxing a mixture of maleic anhydride (0.01 mol), benzenesulfonamide (0.02 mol) and POCl3 for 3hr on a water bath. The reaction mixture was allowed to cool. Ether was added to it. The solid product obtained was filtered off, washed thoroughly with ether and hot alcohol and recrystallized to the constant melting point of 255–259° C

Rod like single crystals used in the X-ray diffraction studies were obtained from a solution of the compound in DMF.

### Refinement

The H atom of the NH group was located in difference map and later restrained to N—H = 0.86 (1) Å. The other H atoms were positioned with idealized geometry using a riding model with C—H = 0.93 Å. All H atoms were refined with isotropic displacement parameters  set to 1.2 times of the *U*_eq_ of the parent atom.

### Crystal data

**Table:**

C_16_H_14_N_2_O_6_S_2_	*F*(000) = 408
*M**_r_* = 394.41	*D*_x_ = 1.535 Mg m^−^^3^
Monoclinic, *P*2_1_/*c*	Mo *K*α radiation, λ = 0.71073 Å
Hall symbol: -P 2ybc	Cell parameters from 1915 reflections
*a* = 8.582 (1) Å	θ = 2.8–27.7°
*b* = 5.1464 (6) Å	µ = 0.35 mm^−^^1^
*c* = 19.691 (4) Å	*T* = 299 K
β = 101.17 (2)°	Rod, colourless
*V* = 853.2 (2) Å^3^	0.48 × 0.28 × 0.22 mm
*Z* = 2	

### Data collection

**Table:**

Oxford Diffraction Xcalibur diffractometer with a Sapphire CCD detector	1720 independent reflections
Radiation source: fine-focus sealed tube	1500 reflections with *I* > 2σ(*I*)
graphite	*R*_int_ = 0.009
Rotation method data acquisition using ω and phi scans	θ_max_ = 26.4°, θ_min_ = 3.5°
Absorption correction: multi-scan (*CrysAlis RED*; Oxford Diffraction, 2009)	*h* = −9→10
*T*_min_ = 0.850, *T*_max_ = 0.927	*k* = −6→6
3236 measured reflections	*l* = −24→14

### Refinement

**Table:**

Refinement on *F*^2^	Primary atom site location: structure-invariant direct methods
Least-squares matrix: full	Secondary atom site location: difference Fourier map
*R*[*F*^2^ > 2σ(*F*^2^)] = 0.030	Hydrogen site location: inferred from neighbouring sites
*wR*(*F*^2^) = 0.083	H atoms treated by a mixture of independent and constrained refinement
*S* = 1.07	*w* = 1/[σ^2^(*F*_o_^2^) + (0.0402*P*)^2^ + 0.3647*P*] where *P* = (*F*_o_^2^ + 2*F*_c_^2^)/3
1720 reflections	(Δ/σ)_max_ = 0.004
121 parameters	Δρ_max_ = 0.25 e Å^−^^3^
1 restraint	Δρ_min_ = −0.32 e Å^−^^3^

### Special details

**Table:**

Experimental. CrysAlis RED (Oxford Diffraction, 2009) Empirical absorption correction using spherical harmonics, implemented in SCALE3 ABSPACK scaling algorithm.
Geometry. All e.s.d.'s (except the e.s.d. in the dihedral angle between two l.s. planes) are estimated using the full covariance matrix. The cell e.s.d.'s are taken into account individually in the estimation of e.s.d.'s in distances, angles and torsion angles; correlations between e.s.d.'s in cell parameters are only used when they are defined by crystal symmetry. An approximate (isotropic) treatment of cell e.s.d.'s is used for estimating e.s.d.'s involving l.s. planes.
Refinement. Refinement of *F*^2^ against ALL reflections. The weighted *R*-factor *wR* and goodness of fit *S* are based on *F*^2^, conventional *R*-factors *R* are based on *F*, with *F* set to zero for negative *F*^2^. The threshold expression of *F*^2^ > σ(*F*^2^) is used only for calculating *R*-factors(gt) *etc*. and is not relevant to the choice of reflections for refinement. *R*-factors based on *F*^2^ are statistically about twice as large as those based on *F*, and *R*- factors based on ALL data will be even larger.

### Fractional atomic coordinates and isotropic or equivalent isotropic displacement parameters (Å^2^)

**Table:**

	*x*	*y*	*z*	*U*_iso_*/*U*_eq_	
C1	0.81684 (18)	0.2173 (3)	0.31947 (8)	0.0310 (3)	
C2	0.7033 (2)	0.0409 (3)	0.28846 (9)	0.0406 (4)	
H2	0.6715	−0.0947	0.3139	0.049*	
C3	0.6381 (2)	0.0704 (4)	0.21881 (10)	0.0539 (5)	
H3	0.5618	−0.0465	0.1971	0.065*	
C4	0.6857 (3)	0.2717 (4)	0.18171 (10)	0.0548 (5)	
H4	0.6417	0.2894	0.1349	0.066*	
C5	0.7979 (3)	0.4475 (4)	0.21310 (11)	0.0539 (5)	
H5	0.8287	0.5837	0.1876	0.065*	
C6	0.8651 (2)	0.4217 (4)	0.28269 (10)	0.0438 (4)	
H6	0.9410	0.5393	0.3043	0.053*	
C7	0.64152 (19)	0.3130 (3)	0.45450 (8)	0.0351 (4)	
C8	0.57352 (19)	0.5096 (3)	0.49559 (8)	0.0370 (4)	
H8	0.6360	0.6481	0.5153	0.044*	
N1	0.79828 (16)	0.3588 (3)	0.45084 (8)	0.0367 (3)	
H1N	0.842 (2)	0.498 (3)	0.4667 (10)	0.044*	
O1	1.05692 (13)	0.3007 (3)	0.42044 (7)	0.0460 (3)	
O2	0.88967 (16)	−0.0871 (2)	0.42525 (7)	0.0474 (3)	
O3	0.56818 (16)	0.1304 (3)	0.42616 (7)	0.0559 (4)	
S1	0.90468 (4)	0.17803 (8)	0.40734 (2)	0.03339 (14)	

### Atomic displacement parameters (Å^2^)

**Table:**

	*U*^11^	*U*^22^	*U*^33^	*U*^12^	*U*^13^	*U*^23^
C1	0.0308 (7)	0.0305 (8)	0.0315 (8)	0.0033 (6)	0.0056 (6)	−0.0026 (6)
C2	0.0435 (9)	0.0360 (9)	0.0399 (9)	−0.0037 (7)	0.0019 (7)	−0.0043 (7)
C3	0.0564 (11)	0.0548 (12)	0.0431 (10)	0.0011 (10)	−0.0087 (9)	−0.0103 (9)
C4	0.0620 (12)	0.0657 (13)	0.0339 (10)	0.0213 (11)	0.0023 (9)	0.0009 (9)
C5	0.0598 (12)	0.0549 (12)	0.0502 (12)	0.0122 (10)	0.0184 (10)	0.0169 (10)
C6	0.0421 (9)	0.0382 (9)	0.0514 (11)	−0.0007 (8)	0.0100 (8)	0.0044 (8)
C7	0.0379 (8)	0.0382 (9)	0.0295 (8)	−0.0074 (7)	0.0070 (6)	−0.0050 (7)
C8	0.0398 (8)	0.0395 (9)	0.0315 (8)	−0.0077 (7)	0.0061 (7)	−0.0077 (7)
N1	0.0354 (7)	0.0335 (7)	0.0412 (8)	−0.0081 (6)	0.0077 (6)	−0.0133 (6)
O1	0.0306 (6)	0.0568 (8)	0.0481 (7)	−0.0031 (6)	0.0013 (5)	−0.0122 (6)
O2	0.0653 (8)	0.0329 (6)	0.0403 (7)	0.0037 (6)	0.0008 (6)	0.0023 (5)
O3	0.0493 (7)	0.0586 (9)	0.0639 (9)	−0.0232 (7)	0.0212 (7)	−0.0315 (7)
S1	0.0335 (2)	0.0318 (2)	0.0329 (2)	0.00033 (16)	0.00156 (15)	−0.00477 (16)

### Geometric parameters (Å, °)

**Table:**

C1—C2	1.384 (2)	C6—H6	0.9300
C1—C6	1.385 (2)	C7—O3	1.206 (2)
C1—S1	1.7600 (16)	C7—N1	1.381 (2)
C2—C3	1.386 (3)	C7—C8	1.484 (2)
C2—H2	0.9300	C8—C8^i^	1.310 (3)
C3—C4	1.375 (3)	C8—H8	0.9300
C3—H3	0.9300	N1—S1	1.6541 (15)
C4—C5	1.378 (3)	N1—H1N	0.840 (9)
C4—H4	0.9300	O1—S1	1.4288 (12)
C5—C6	1.386 (3)	O2—S1	1.4215 (13)
C5—H5	0.9300		
			
C2—C1—C6	121.56 (16)	C5—C6—H6	120.6
C2—C1—S1	119.29 (13)	O3—C7—N1	122.36 (16)
C6—C1—S1	119.14 (13)	O3—C7—C8	123.95 (15)
C1—C2—C3	118.68 (18)	N1—C7—C8	113.69 (14)
C1—C2—H2	120.7	C8^i^—C8—C7	120.7 (2)
C3—C2—H2	120.7	C8^i^—C8—H8	119.6
C4—C3—C2	120.25 (19)	C7—C8—H8	119.6
C4—C3—H3	119.9	C7—N1—S1	124.88 (11)
C2—C3—H3	119.9	C7—N1—H1N	119.4 (13)
C3—C4—C5	120.70 (18)	S1—N1—H1N	115.1 (13)
C3—C4—H4	119.7	O2—S1—O1	120.19 (8)
C5—C4—H4	119.7	O2—S1—N1	109.02 (8)
C4—C5—C6	120.08 (19)	O1—S1—N1	103.61 (7)
C4—C5—H5	120.0	O2—S1—C1	108.18 (8)
C6—C5—H5	120.0	O1—S1—C1	109.22 (8)
C1—C6—C5	118.74 (18)	N1—S1—C1	105.68 (7)
C1—C6—H6	120.6		
			
C6—C1—C2—C3	0.5 (3)	C8—C7—N1—S1	−178.46 (12)
S1—C1—C2—C3	−178.39 (14)	C7—N1—S1—O2	−49.95 (17)
C1—C2—C3—C4	−0.1 (3)	C7—N1—S1—O1	−179.05 (14)
C2—C3—C4—C5	−0.4 (3)	C7—N1—S1—C1	66.13 (16)
C3—C4—C5—C6	0.5 (3)	C2—C1—S1—O2	23.00 (16)
C2—C1—C6—C5	−0.3 (3)	C6—C1—S1—O2	−155.88 (14)
S1—C1—C6—C5	178.50 (14)	C2—C1—S1—O1	155.46 (13)
C4—C5—C6—C1	−0.1 (3)	C6—C1—S1—O1	−23.41 (16)
O3—C7—C8—C8^i^	1.4 (3)	C2—C1—S1—N1	−93.64 (14)
N1—C7—C8—C8^i^	−179.2 (2)	C6—C1—S1—N1	87.48 (14)
O3—C7—N1—S1	1.0 (3)		

Symmetry codes: (i) −*x*+1, −*y*+1, −*z*+1.

### Hydrogen-bond geometry (Å, °)

**Table:**

*D*—H···*A*	*D*—H	H···*A*	*D*···*A*	*D*—H···*A*
N1—H1N···O2^ii^	0.84 (1)	2.35 (2)	3.0254 (19)	138 (2)
N1—H1N···O1^iii^	0.84 (1)	2.45 (2)	3.1335 (19)	139 (2)

Symmetry codes: (ii) *x*, *y*+1, *z*; (iii) −*x*+2, −*y*+1, −*z*+1.
