# *N*,*N*′-Bis(phenyl­sulfon­yl)maleamide. Corrigendum

**DOI:** 10.1107/S1600536810009025

**Published:** 2010-04-14

**Authors:** B. Thimme Gowda, Sabine Foro, P. A. Suchetan, Hartmut Fuess

**Affiliations:** a Department of Chemistry, Mangalore University, Mangalagangotri 574 199, Mangalore, India; b Institute of Materials Science, Darmstadt University of Technology, Petersenstrasse 23, D-64287 Darmstadt, Germany

## Abstract

The chemical name of the title compound in the paper by Gowda, Foro, Suchetan & Fuess [*Acta Cryst.* (2010), E**66**, o187] is corrected.

## Abstract 2

Corrigendum to *Acta Cryst.* (2010), E**66**, o187.

In the paper by Gowda *et al.* (2010) ▶, the chemical name given in the *Title* should be for the *trans* rather than the *cis* isomer, *i.e.* the title should be ‘*N*,*N*′-Bis(phenyl­sulfon­yl)fumar­amide’.
